# Frequency Dependant Topological Alterations of Intrinsic Functional Connectome in Major Depressive Disorder

**DOI:** 10.1038/srep09710

**Published:** 2015-04-09

**Authors:** Qinghua Luo, Zhou Deng, Jingxue Qin, Dongtao Wei, Lingli Cun, Jiang Qiu, Glen Hitchman, Peng Xie

**Affiliations:** 1 Department of psychiatry, The First Affiliated Hospital of Chongqing Medical University, Chongqing 400016, China; 2 Key laboratory of cognition and personality (SWU), Ministry of Education, Chongqing 400715, China; 3 Department of psychology, Southwest University, Chongqing 400715, China; 4 Department of Neurology, The First Affiliated Hospital of Chongqing Medical University, Chongqing 400016, China; 5 Institute of Neuroscience and the Collaborative Innovation Center for Brain Science, Chongqing Medical University, Chongqing 400016, China

## Abstract

Major depressive disorder is associated with aberrant topological organizations of brain networks. However, whether this aberrance is shown in broader frequency bands or in a specific frequency band remains unknown. Fifty patients and fifty gender, age and education matched normal controls underwent resting state functional magnetic resonance imaging. Frequency dependent topological measures based on graph theory were calculated from wavelet decomposed resting state functional brain signals. In the specific frequency band of 0.03–0.06 14Hz, the clustering coefficient and the global efficiency were reduced while the characteristic path length was increased. Furthermore, patients showed aberrant nodal centralities in the default mode network, executive network and occipital network. Network based statistical analysis revealed system-wise topological alterations in these networks. The finding provides the first systematic evidence that depression is associated with frequency specific global and local topological disruptions and highlights the importance of frequency information in investigating major depressive disorders.

Major depressive disorder (MDD) is among the most common forms of psychiatric disease with a world-wide prevalence of 16% across the lifespan and 6.6% across a 12 month period^1^. Core diagnostic criteria include, but are not limited to, persistent and pervasive sadness, an inability to concentrate, anhedonia and irritability^2^. In the year 2000, the World Health Organization declared MDD as being the fourth biggest contributor to the burden of disease worldwide and predicted is to become the second biggest contributor by 2020^3^.

A recent review of MDD found replicable grey matter alterations in the frontal cortex, cingulate cortex, orbitalfrontal cortex, hippocampus and straitum^4^. A meta-analytic review of diffusion tensor imaging (DTI) studies found consistent decreased fractional anisotropy in the bilateral frontal cortex, right fusiform gyrus and right occipital cortex^5^. Evidence from functional magnetic resonance imaging (fMRI) studies also points to abnormal activity in the frontal lobe^6^, insular cortex^7^, temporal lobe^8^, occipital lobe^9^ and subcortical structures (amygdale and straitum in particular). A possible and plausible interpretation of these wide regional alterations is that depression is associated with aberrant coupling between these anatomical regions, which is supported by functional connectivity studies of MDD^10^,^11^,^12^.

Recent research has highlighted the benefits of graph theory based analysis of human brain networks^13^. Previous studies suggested the human brain exhibits an organization principle of small worldness (i.e. being highly segregated and integrated) across various modalities^14^,^15^,^16^. Moreover, the configuration of brain connectomes in MDD patients was disrupted^17^,^18^,^19^,^20^, though the results were mixed. Previous studies have highlighted the importance of frequency information in neural oscillations^21^,^22^ and resting state functional MRI signals^23^,^24^,^25^. Frequency dependent graphical analysis may help better understand the pathological brain mechanisms associated with depression. As we know, wavelet analysis decomposes the signal into several scales (frequency bands) and is more effective in handling signals with the property of fractional scaling^26^. Wavelet decomposition had been used in characterizing networks across many mental diseases such as schizophrenia^27^, Alzheimer's disease^24^ and amnesic mild cognitive impairment^25^. With respect to MDD, only one study used wavelet analysis to investigate brain network configurations in MDD patients^28^. However, this study by Manoliu and his colleagues^28^ only investigated a specific frequency band of 0.060 ~ 0.125 14Hz, which may have overlooked valuable information in other frequency bands.

Here we measured functional connectivity and examined topological organization of MDD patients in resting state fMRI (R-fMRI) data. In brief, processing procedures included (1) constructing network matrices based on wavelet decomposed R-fMRI data, (2) calculating network topological metrics, (3) comparing topological metrics across groups for each wavelet scale and (4) correlating topological metrics with clinical variables. Given previous graph theory based studies of MDD^17^,^18^,^19^,^20^,^28^, We hypothesized that the brain networks of MDD patients would be disrupted at both global and regional levels. A recent study showed that intelligence was mediated by the coupling of brain networks across several frequency bands^29^. We thus also hypothesized that the aberrant brain networks of MDD patients may be manifestated across multiple frequency intervals.

## Results

### Demographic statistics

The depression group and healthy control (HC) group were matched in gender, age and education (all p > 0.05). The two groups significantly differed in HRSD scores (p < 0.001). Comprehensive demographic results are listed in Table 1.

### Frequency specific global topological alterations in patients

**Figure 1:**
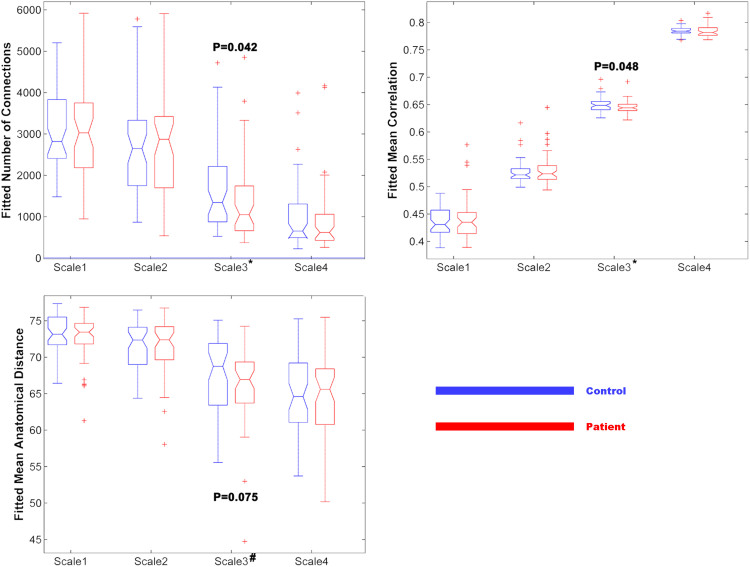
Group comparisons of fitted number of connections, fitted mean correlation strength and fitted mean anatomical distance (mean Euclidean distance across existing edges) across four wavelet scales. * denotes that p < 0.05; # denotes that 0.05 < p < 0.1.

**Figure 2:**
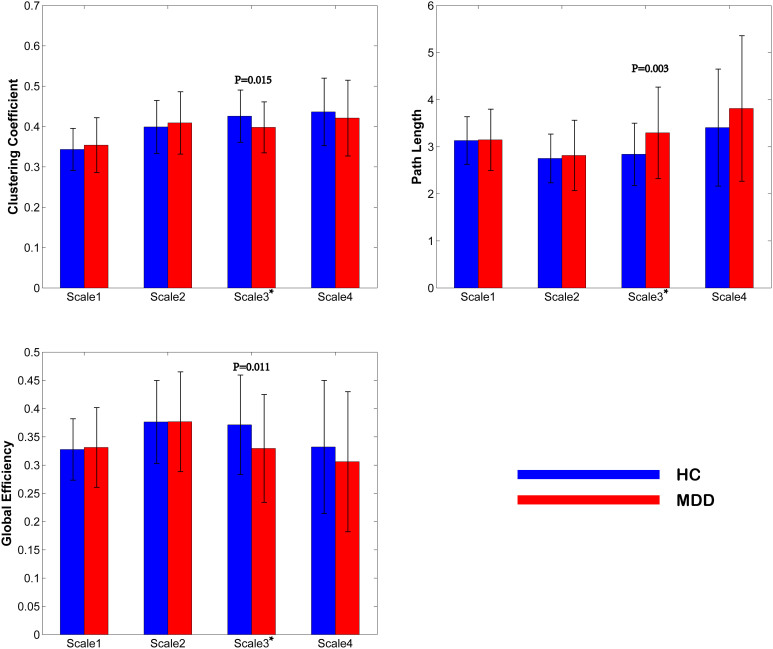
Group comparisons of global topological metrics across four wavelet scales. Here, only topological metrics which show significant group differences are presented. These metrics include clustering coefficient (upper left), characteristic path length (upper right) and global efficiency (lower left). * denotes that p < 0.05; # denotes that 0.05 < p < 0.1.

Significant group differences in global topology were exclusively found in Scale 3 (0.03–0.06 14Hz). First, in the specific thresholded network of Scale 3, patients and HCs differed significantly in the total number of connections and the mean correlation as well as the mean anatomical distance (mean Euclidean distance across existing edges). The patient group featured less connections, weaker connections and shorter anatomical connections (mathematically defined as the Euclidean distance between centroids of every two nodes) in Scale 3 (See Figure 1). Second and further, as shown in Figure 2, global topological metrics exhibited significant alterations in Scale 3 but not in the other Scales. In patients, the clustering coefficient and the global efficiency were reduced while the characteristic path length was increased.

### Aberrant nodal centrality

**Figure 3:**
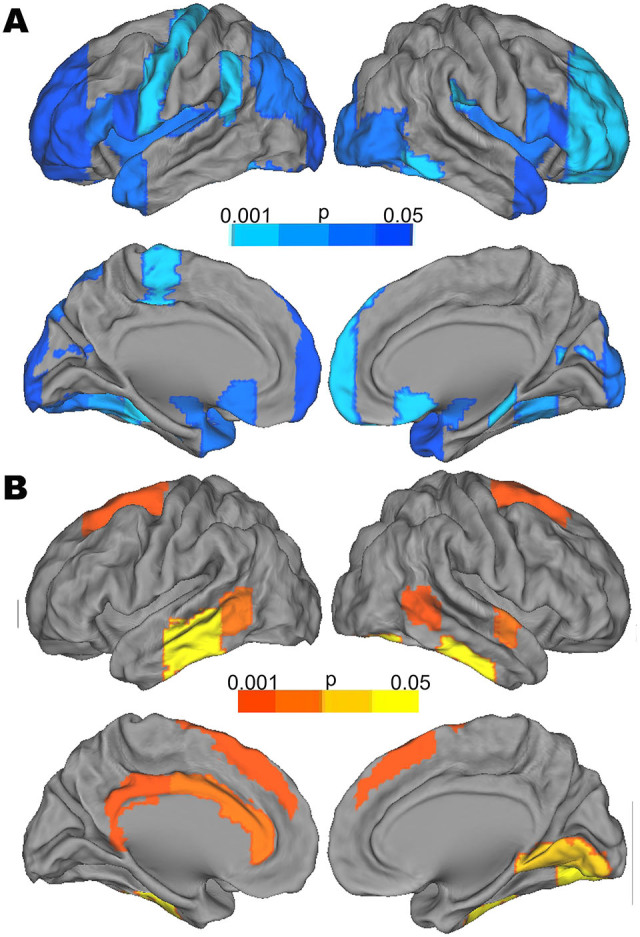
Regions which show significant decreased degree centrality (Upper panel, A) and increased betweenness centrality (Lower panel, B) in patients. CARET software (CARET; http://brainvis.wustl.edu) was used for surface rendering. The cold color represents that the degree centrality of a given region was significantly (p < 0.05, uncorrected) lower in MDD patients. The hot color represents that the betweenness centrality of a given region was significantly (p < 0.05, uncorrected) lower in MDD patients. The color bar indicates p value;

Degree centrality was altered (p < 0.05 uncorrected) in several cortical and subcortical regions (see Figure 3 and Table 2). Patients had decreased degree centrality (i.e. less connections) predominately in nodes relevant to executive function, emotion processing and basic sensory function. They also showed increased betweenness centrality in cortical midline structures (CMS), which are part of the default mode network and are a core component of self processing.

### Aberrant functional connectivity

**Figure 4:**
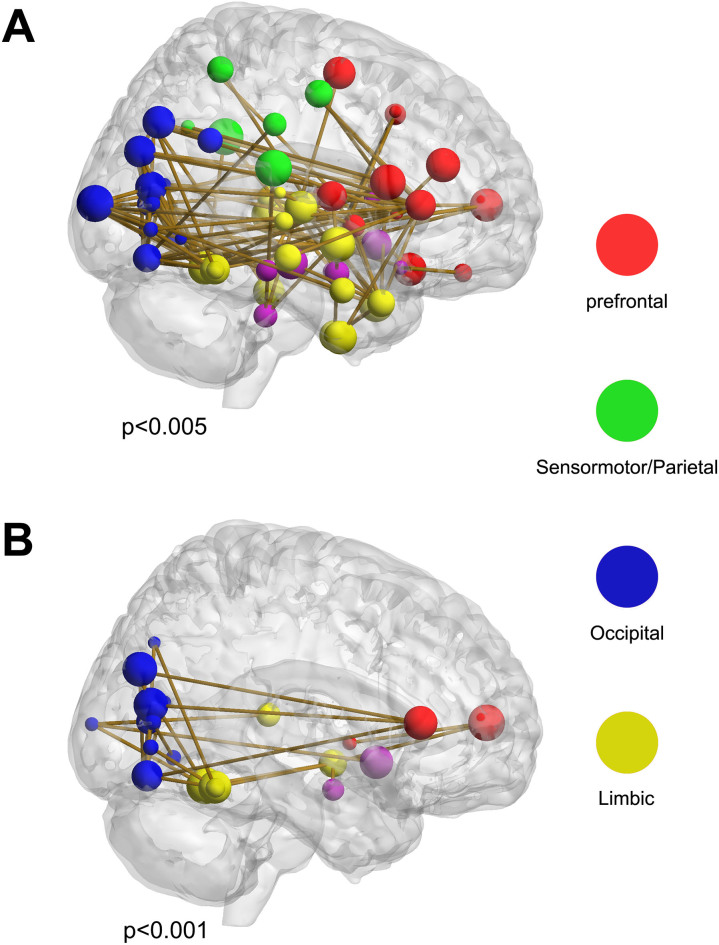
Networks with decreased connections in patients. (A) The larger network identified by the network based statistical (NBS) analysis method under the liberal primary threshold of p < 0.005; (B) The smaller network identified by NBS under the strict primary threshold of p < 0.001; BraiNet Viewer (http://www.nitrc.org/projects/bnv/) was used for visualization.

For a liberal primary threshold of p = 0.005, the NBS method detected a dysconnected network (p < 0.05. corrected) comprised of 136 decreased connections linking wide spread regions (see Figure 4A, Table 3 lists the nodes). Under a strict threshold of p = 0.001, the NBS method indentified a smaller network comprised of 23 reduced functional connections in patients (see Figure 4B, Table 3 lists the nodes). Of note, networks identified by the NBS method can only be statistically significant as a whole. Any connections within cannot be declared as independently significant^30^.

### Correlation between topological metrics and clinical variables

**Figure 5:**
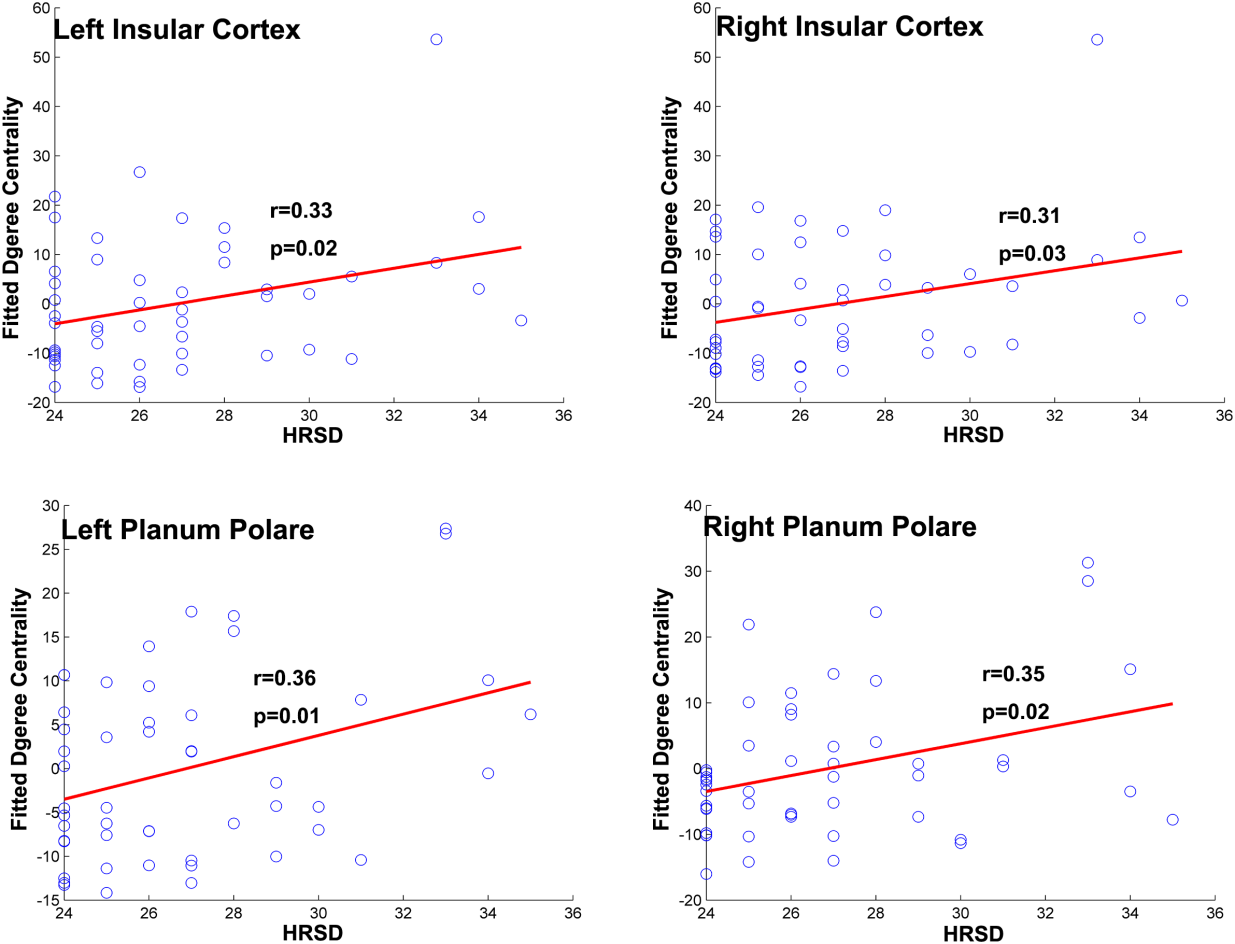
Regions showing significant correlations between nodal centrality and HRSD score. Fitted degree centrality was calculated by regressing out nuisance variables. HRSD: Hamilton Rating Scale for Depression.

None of the global topological metrics significantly covaried with HRSD score or disease duration. Among the regions showing significant group differences, the bilateral insular cortex and bilateral planum polare exhibited trend of positive correlations with HRSD scores (see Figure 5), but they did not survive multiple corrections.

## Discussion

The present study examined the topological organization of the brain networks of MDD patients in R-fMRI data and found topological alterations in the specific and most salient wavelet scale of 0.03 ~ 0.06 14Hz^14^. Globally, we observed a reduction in the clustering coefficient and global efficiency as well as an increase in characteristic path lengths. Regionally, we found aberrant nodal centralities in widely distributed cortical and subcortical regions. Finally, the NBS method identified dysconnected subsystems (under primary thresholds of 0.005 and 0.001 respectively) in patients.

Significant global topological differences between networks of MDD patients and HCs were only found in the specific frequency band of 0.03 ~ 0.06 14Hz. Previous researchers have suggested that neuronal oscillations are linearly arranged on the natural logarithmic scale and this regularity indicates that different frequency bands result from different oscillators with different biological properties and functions^21^,^22^. Although the exact mechanisms underlying signals of different frequency bands remains poorly understood, the 0.03 ~ 0.06 14Hz range proved to be most salient resting brain state in normal adults^14^. Moreover, disrupted topological architectures of amnestic mild cognitive impairment (aMCI) patients^25^ and Alzheimer's disease (AD) patients^24^ as well as regional alterations of Attention deficit hyperactivity disorder (ADHD) patients^23^ were in this specific frequency band. A recent work highlight intelligence of healthy adults was mediated by coupling between multiple networks across multiple frequencies^29^, This evidence in conjunction with the present results and previous studies might serve to highlight the clinical significance of brain oscillations under 0.03 ~ 0.06 14Hz.

The human brain functions like a small world^14^. In the powerful framework of graph theory, a network is declared as small world when it attains a perfect balance between local specialization (indexed by a high clustering coefficient) and global integration (indexed by low characteristic path length)^13^. The present study found the brain networks of MDD patients and HCs both exhibited the organization principles of small worldness.

Though networks of MDD patients preserve small worldness, the configurations of networks in MDD patients were perturbed. First, networks of MDD patients demonstrated fewer connections and weaker connections. Compatible with our finding in the R-fMRI data, studies based on DTI data also indicated sparse and reduced white matter connectivity in MDD patients^31^. Second, compared to HCs, the clustering coefficient and global efficiency of networks in MDD patients were reduced while characteristic path length was increased. This suggests that the architecture of networks in patients were less locally specialized and less globally integrated.

Degree centrality was decreased in 38 regions of MMD patients' brain networks. Most of these regions were relevant to cognitive executive functions, emotion processing and basic sensory functions. Within the executive control network (which mainly involves the lateral frontal and superior parietal lobules)^32^, the present study indentified fewer global connections in the frontal pole, inferior frontal gyrus and posterior superamarginal gyrus. This may suggest that patients were less skilled in executive functioning as supported by working memory^33^ and Go/Nogo studies^34^. With respect to emotion processing, researchers have documented impaired prefrontal-limbic circuits in patients with MDD^35^. Compatible with these results, we found decreased degree centrality in the prefrontal cortex and limbic areas. Specifically, some researchers have underlined the pathophysiological role of the subcallosal cingulate gyrus and amygdala in depression^36^,^37^. The observed reduced degree centrality in the prefrontal-limbic circuit may be attributable to inadequate reciprocal interactions between the prefrontal cortex and limbic areas. Finally, patients with MDD were found to have low concentrations of γ- aminobutyric acid (GABA)^38^ and efficient treatment brings GABA to presymptomatic levels^39^. The observed reduction of occipital regions may result from disruptions to the metabolism of the occipital cortex in MDD patients.

Betweenness centrality was increased mainly in cortical midline structures (CMS)^40^. CMS refer to brain structures situated near the medial wall of the brain and is mainly comprised of the ventral medial prefrontal cortex, dorsal medial prefrontal cortex, anterior cingulate cortex (the supragenual part in particular) and posterior cingulate cortex. CMS are core biological components for the self^40^. Depression is characterized by a stronger tendency toward introspecting and self-focused cognitions^41^. Hyperactivity in CMS has been recorded in MDD patients^42^. Therefore, the elevated betweenness centrality in patients may reflect the strengthened communication among CMS regions and constitute the pathophysiological basis of rumination in MDD patients.

The NBS method was implemented to locate aberrant connections in MDD patients. A larger disconnected network was identified under the liberal threshold (p = 0.005). Most nodes of this network were those nodes with altered nodal centrality. Furthermore, when we employed a strict threshold (p = 0.001), a smaller abnormal network featuring a majority of long-range connections was found. This suggests that deficient global information integration in patients was mainly driven by decreased long-range connectivity. Of note, this smaller network included nodes within the prefrontal-limbic circuit and provides empirical support for the proposed pivotal role of the prefrontal-limbic circuit in the pathophysiology of depression^35^,^36^,^37^.

Of additional note, the disease duration of the patients here were above 4 years, which means that the current finding may reflect the chronic aspects of depression disorder. Previous psychological studies already indicated that chronically depressed patients differed from acute depressed patients in cognitive functions^43^, response to medications^44^. However, we find few studies investigated the aberrant brain mechanisms of medication free chronic depression. Therefore, the present finding may help gain insight into this particular field and future neuroimaging studies which compared chronically depressed patients with acute ones may further shed lights to the specific brain dysfunction of chronically ones.

The present study is not without limits. First, patients in our study included 17 medicated patients. Some researchers reported a normalization influence of depression treatments on functional connectivity^45^,^46^. However, the exact mechanism remains largely unknown^47^. Moreover, we think the present results were not likely to have been driven by medication effects for the following reasons: (1) medication status (0 or 1) did not significantly covary with any global topological metrics (all p > 0.05); (2) global topological metrics did not significantly differ between medicated and non-medicated patients; (3) when we regressed out medication status, similar results were obtained (i.e. significant global topological differences were exclusively found in wavelet scale. Second, the significances of group comparisons in nodal centrality did not survive stringent bonferroni or liberal fdr corrections. Therefore, the results should be viewed as an explorative attempt and could be reaffirmed by methods with more statistical power (larger samples or selecting prior regions of interest). Third, methodological selections such as parcellation schemes, threshoding methods, smoothing effects, network types (binary or weighted) and modality types may have impacted upon our findings^48^. This may partly account for the discrepancy between finding across network based studies of MDD patients^17^,^18^,^19^,^20^,^28^.

To our knowledge, our work is the first to explore frequency dependent topological alterations in major depressive disorder. Compared to age, gender and education level matched controls, patients showed both global and regional topological alterations in the specific and most salient frequency range of 0.03 ~ 0.06 14Hz. The finding provides the first systematic evidence that depression is associated with frequency specific global and local topological disruptions and highlights the importance of frequency information in investigating major depressive disorders.

## Methods

### Participants

Fifty patients diagnosed with major depressive disorder and fifty normal controls participated in this investigation. No participants were left-handed as measured by the Edinburgh Handedness Inventory^49^. Written informed consent was obtained before the experiment began. Patients were recruited from Chongqing Medical School and diagnosed by an experienced psychiatrist according to the Diagnostic and Statistical Mannual of Mental Disorders-IV. The severity of depression symptoms was assesed by the Hamilton Rating Scale for Depression (HRSD)^50^. All patients had a minimun HRSD score of 18. Nineteen patients had been receiving antidepressant therapy, but were free of any treatment 4 weeks before recruiment. Normal controls were recuited from the local community around Southwest University, Chongqing. All controls scored less than 7 on the HRSD. No participants met the exlusion criteria of any neurological or mental disorder besides depreesion, substance abuse or head trauma. The principls of the present study was adequately guided the approved guidelines which was in accordance with the Decleartion of Helsinki. The study was approved by the Southwest University Brain Imaging Center Institutional Review Board.

### Image Acquisition

All images were acquired on a 3.0-T Siemens Trio MRI scanner using a 32-channel whole-brain coil (Siemens Medical, Erlangen, Germany). High-resolution T1-weighted 3D images were acquired using a magnetization-prepared rapid gradient echo (MPRAGE) sequence (echo time (TE) = 2.52 14ms; repetition time (TR) = 1900 14ms; inversion time (TI) = 900 14ms; flip angle = 9 degrees; slices = 176; thickness = 1.0 14mm; resolution matrix = 256 × 256; voxel size = 1 × 1 × 1 14mm^3^). For each participant, 242 functional images were acquired with a gradient echo type Echo Planar Imaging (EPI) sequence (echo time (TE) = 30 14ms; repetition time (TR) = 2000 14ms; flip angle = 90 degrees; slices = 32; slice thickness = 3.0 14mm; slice gap = 1 14mm; resolution matrix = 64 × 64; voxel size = 3.4 × 3.4 × 4 14mm^3^). During image acquisition, participants were instructed to keep their eyes closed while keeping their head as still as possible without falling asleep. All participants stayed awake during MRI examination which was confirmed by the participant after the examination.

### Data preprocessing

Data preprocessing was performed using DPARSF (http://restfmri.net). The first 10 EPI scans were discarded to suppress equilibration effects. The remaining 232 scans of each subject underwent slice timing correction by sinc interpolating volume slices, motion correction for volume to volume displacement, spatial normalization to standard Montreal Neurological Institute (MNI) space using affine transformation and nonlinear deformation with a voxel size of 3 × 3 × 3 14mm^3^, temporal high pass filtering (≥0.01 14Hz) to reduce the effect of low frequency drift, and regressing out 24 head-motion parameters (6 motion parameters for current the volume, 6 motion parameters for the previous volume and 12 corresponding squared items). Originally proposed by Friston and his colleagues^51^, the Friston 24 parameter model outperformed other motion correction strategies in reducing motion related effects in a recent work^52^. Furthermore, no subjects were found to have excess head motion (translation > 1 14mm or rotation > 1) and head motion profiles were statistically matched in any direction (all p > 0.05) between groups. Given the debate over removing global signal in R-fMRI data preprocessing^53^,^54^, we did not regress the global signal as had been done in previous wavelet studies^14^,^24^,^27^. To exclude cofounding effect from CSF and white matter signals, we further regressed out the CSF and WM signals. A custom grey matter mask was created by thresholding the mean GM probability map from all individuals (threshold = 0.2). This procedure was implemented in SPM 8 (http://www.fil.ion.ucl.ac.uk/spm/software/spm8/). All subsequent analyses were restricted to the custom grey matter mask.

### Network construction

#### Node definition

Nodes were obtained by anatomically dividing the brain into 112 distinct regions according to the Harvard-Oxford atlas^55^.

#### Edge definition

First, the mean time series was extracted from each region. Second, the maximal overlap discrete wavelet transform (MODWT) method^56^ was used to decompose each regional mean R-fMRI time series into four successive scales or frequency intervals (Scale 1, 0.12–0.25 14Hz; Scale 2 0.06–0.12 14Hz; Scale 3, 0.03–0.06 14Hz; Scale 4, 0.015–0.03 14Hz). Third, interregional pairwise Pearson correlations and their corresponding statistical significance levels (i.e. p values) were computed for each of the four scales. This resulted in four frequency-specific correlation matrices and p-matrices for each subject.

#### Network threshold

To exclude possible spurious edges, we employed a rigorous Bonferroni correction (p < 0.05, corrected) for each of the four correlation matrices. Namely, only edges with a corrected p value smaller than 0.05 were retained^57^.

### Network analysis

For each subject, the undirected weighted matrix was used for network analysis. First, global topological metrics were calculated across all four wavelet scales. The wavelet scales for which patients demonstrate abnormal global topological metrics were identified and chosen for further analysis. Global topological metrics included the clustering coefficient^58^, characteristic path length^58^, small worldness^58^, global efficiency^59^. Second, for the selected wavelet scales, nodal centrality was calculated for each node and compared across groups. Here, nodal centrality refers to degree centrality and betweenness centrality^59^. Regional analyses were exploratory to get a complete understanding of nodal aberrancies. All topological metrics were automatically computed by GRETNA (https://www.nitrc.org/projects/gretna/). Detailed definitions of topological metrics can be found in the Supplementary Materials section.

### Statistical analysis

#### Group comparisons

Group comparisons of topological metrics (global and regional) were carried out by permutation tests (10000 permutations) while controlling for gender, age and education. Briefly, for each permutation, subjects were randomly shuffled and the group difference of a given metric was re-computed. We obtained a null distribution of group differences of that metric and the significance level of the real group difference was determined by the percentage of permutations with a group difference equal to or greater than the real group difference in all permutations (10000). In order to find aberrant connections in patients, we carried out a method called network based statistical (NBS) analysis^30^. Specifically, first, edges with a p value (obtained by a two sample t-test) less than a primary uncorrected threshold (p < 0.005 and p < 0.001 respectively) were identified. Based on these superathreshold edges, several components or subnetworks were then identified and their sizes (number of edges within a subnetwork) calculated. Second, the significance level of each identified components was computed according to a null distribution of the largest subnetwork size which was empirically obtained through 10000 permutations. The significance level of any originally found subnetwork (i.e. before permutation test) with size M was computed as the percentage of permutations in which the size of the largest component exceeded or equaled M. Only significant subnetworks (p < 0.05, family wise error corrected) were reported. The reason we use two primary threshold (here, p < 0.005 and p < 0.001 respectively) is to give a full understanding of aberrant connections in patients as done in previous studies^25^,^60^. In addition, we use fdr (p < 0.05 corrected) here for multiple comparisons (2 comparisons here: p < 0.005 and p < 0.001).

#### Correlation analysis between topological metrics and clinical variables

Partial correlation analysis was employed to assess the relationships between topological metrics and clinical variables (HRSD scores and disease durations) while regressing out nuisance variables (i.e. gender, age, education).

## Supplementary Material

Supplementary Information Definition of topological metrics

## Figures and Tables

**Table 1:** Demographic and clinical data

	HC	MDD	p value
Subjects	50	50	
Gender	26 males	23 males	>0.05^b^
Age	40.02 ± 11.74	41.6 ± 12.16	>0.05^a^
Education level (years)	11.18 ± 3.46	11.18 ± 3.89	>0.05^a^
HRSD score	2.12 ± 1.92	26.88 ± 3.06	<0.001^a^
Duration of depression (months)		48.61 ± 65.39	

Data were presented as mean ± SD;

HC, Health control; MDD, Major depression disorder; HRSD, Hamilton Depression Rating Scale;

^a^The p value was obtained by two-sample t test;

^b^The p value was obtained by pearson chi-square test;

**Table 2:** Brain regions showed decreased degree centrality in patients

Lobe	Node (brain region)	Side	p value
Frontal lobe	Frontal Pole	L	0.006
	Frontal Pole	R	0.036
	Inferior Frontal Gyrus, pars triangularis	L	0.023
	Inferior Frontal Gyrus, pars triangularis	R	0.006
	Inferior Frontal Gyrus, pars opercularis	L	0.004
	Inferior Frontal Gyrus, pars opercularis	R	0.023
	Insular Cortex	L	0.018
	Insular Cortex	R	0.02
	Frontal Operculum Cortex	R	0.043
	Subcollosal Cortex	L	0.02
	Subcollosal Cortex	R	0.041
Sensorimotor lobe	Precentral Gyrus	L	0.041
	Central Opercular Cortex	L	0.032
Parietal lobe	Parietal Operculum Cortex	R	0.04
	Superamarginal Gyrus, posterior division	L	0.049
Temporal lobe	Temporal Pole	L	0.013
	Temporal Pole	R	0.01
	Planum Polare	L	0.002
	Planum Polare	R	0.006
	Heschls Gyrus	L	0.015
	Planum Temporale	L	0.016
	Temporal Fusiform Cortex, posterior division	L	0.044
	Temporal Occipital Fusiform Cortex	L	0.031
	Temporal Occipital Fusiform Cortex	R	0.019
	Inferior Temporal Gyrus, temporoocipital part	R	0.046
Occipital lobe	Occipital Fusiform Gyrus	L	0.014
	Occipital Pole	L	0.008
	Occipital Pole	R	0.014
	Lateral Occipital Cortex, inferior division	R	0.017
	Lateral Occipital Cortex, superior division	L	0.017
	Supracalcarine Cortex	L	0.016
	Supracalcarine Cortex	R	0.035
	Cuneual Cortex	R	0.042
Subcortical lobe	Parahippocample Gyrus, posterior division	R	0.039
	Caudate	R	0.045
	Putamen	R	0.044
	Amygdala	L	0.019
	Amygdala	R	0.011

*L, left; R, right.

**Table 3:** Brain regions showed increased betweenness centrality in patients

Lobe	Node (brain region)	Side	p value
Frontal lobe	Superior Frontal Gyrus	L	0.004
	Superior Frontal Gyrus	R	0.003
	Cingulate Cortex, anterior division	L	0.022
	Cingulate Cortex, posterior division	R	0.003
Temporal lobe	Superior Temporal Gyrus, anterior division	R	0.017
	Middle Temporal Gyrus, posterior division	L	0.047
	Middle Temporal Gyrus, temporoccipital part	L	0.022
	Middle Temporal Gyrus, temporoccipital part	R	0.003
	Inferior Temporal Gyrus, posterior division	L	0.034
	Inferior Temporal Gyrus, posterior division	R	0.039
Occipital lobe	Lingual Gyrus	R	0.029
	Occipital Fusiform Gyrus	R	0.036
Subcortical	Parahipocample Gyrus, posterior division	L	0.039

*L, left; R, right.

## References

[b1] Kessler R. C. *et al.* The epidemiology of major depressive disorder. JAMA 289, 3095–3105 (2003). 12813115 10.1001/jama.289.23.3095

[b2] Nestler E. J. *et al.* Neurobiology of depression. Neuron 34, 13–25 (2002). 11931738 10.1016/s0896-6273(02)00653-0

[b3] Guilbert J. J. The world health report 2002 - reducing risks, promoting healthy life. Education for health (Abingdon, England) 16, 230 (2003). 10.1080/1357628031000116808 14741909

[b4] Arnone D., McIntosh A. M., Ebmeier K. P., Munafò M. R. & Anderson I. M. Magnetic resonance imaging studies in unipolar depression: systematic review and meta-regression analyses. Eur. Neuropsychopharmacol. 22, 1–16 (2012). 21723712 10.1016/j.euroneuro.2011.05.003

[b5] Liao Y. *et al.* Is depression a disconnection syndrome? Meta-analysis of diffusion tensor imaging studies in patients with MDD. J. Psychiatry Neurosci. 38, 49–56 (2013). 22691300 10.1503/jpn.110180 PMC3529219

[b6] Ritchey M., Dolcos F., Eddington K. M., Strauman T. J. & Cabeza R. Neural correlates of emotional processing in depression: Changes with cognitive behavioral therapy and predictors of treatment response. J. Psychiatr. Res. 45, 577–587 (2011). 20934190 10.1016/j.jpsychires.2010.09.007 PMC3042483

[b7] Sliz D. & Hayley S. Major depressive disorder and alterations in insular cortical activity: a review of current functional magnetic imaging research. Front Hum Neurosci 6, 323 (2012). 23227005 10.3389/fnhum.2012.00323 PMC3512092

[b8] Grimm S. *et al.* Reduced negative BOLD responses in the default-mode network and increased self-focus in depression. World Journal of Biological Psychiatry 12, 627–637 (2011). 21247256 10.3109/15622975.2010.545145

[b9] Chantiluke K. *et al.* Fronto-striato-cerebellar dysregulation in adolescents with depression during motivated attention. Biol. Psychiatry 71, 59–67 (2012). 22015111 10.1016/j.biopsych.2011.09.005

[b10] Anand A., Li Y., Wang Y., Lowe M. J. & Dzemidzic M. Resting state corticolimbic connectivity abnormalities in unmedicated bipolar disorder and unipolar depression. Psychiatry Res. - Neuroimaging 171, 189–198 (2009). 19230623 10.1016/j.pscychresns.2008.03.012 PMC3001251

[b11] Greicius M. D. *et al.* Resting-State Functional Connectivity in Major Depression: Abnormally Increased Contributions from Subgenual Cingulate Cortex and Thalamus. Biol. Psychiatry 62, 429–437 (2007). 17210143 10.1016/j.biopsych.2006.09.020 PMC2001244

[b12] Irwin W. *et al.* Amygdalar interhemispheric functional connectivity differs between the non-depressed and depressed human brain. Neuroimage 21, 674–686 (2004). 14980569 10.1016/j.neuroimage.2003.09.057

[b13] Sporns O., Tononi G. & Kötter R. The human connectome: A structural description of the human brain. PLoS Computational Biology 1, 0245–0251 (2005). 10.1371/journal.pcbi.0010042 PMC1239902 16201007

[b14] Achard S., Salvador R., Whitcher B., Suckling J. & Bullmore E. A resilient, low-frequency, small-world human brain functional network with highly connected association cortical hubs. J. Neurosci. 26, 63–72 (2006). 16399673 10.1523/JNEUROSCI.3874-05.2006 PMC6674299

[b15] Gong G. *et al.* Mapping anatomical connectivity patterns of human cerebral cortex using in vivo diffusion tensor imaging tractography. Cereb. Cortex 19, 524–536 (2009). 18567609 10.1093/cercor/bhn102 PMC2722790

[b16] He Y., Chen Z. J. & Evans A. C. Small-world anatomical networks in the human brain revealed by cortical thickness from MRI. Cereb. Cortex 17, 2407–2419 (2007). 17204824 10.1093/cercor/bhl149

[b17] Jin C. *et al.* A preliminary study of the dysregulation of the resting networks in first-episode medication-naive adolescent depression. Neurosci. Lett. 503, 105–109 (2011). 21871534 10.1016/j.neulet.2011.08.017

[b18] Leistedt S. J. J. *et al.* Altered sleep brain functional connectivity in acutely depressed patients. Hum. Brain Mapp. 30, 2207–2219 (2009). 18937282 10.1002/hbm.20662 PMC6870637

[b19] Singh M. K. *et al.* Anomalous gray matter structural networks in major depressive disorder. Biol. Psychiatry 74, 777–785 (2013). 23601854 10.1016/j.biopsych.2013.03.005 PMC3805751

[b20] Zhang J. *et al.* Disrupted brain connectivity networks in drug-naive, first-episode major depressive disorder. Biol. Psychiatry 70, 334–342 (2011). 21791259 10.1016/j.biopsych.2011.05.018

[b21] Buzsáki G., Geisler C., Henze D. A. & Wang X. J. Interneuron Diversity series: Circuit complexity and axon wiring economy of cortical interneurons. Trends in Neurosciences 27, 186–193 (2004). 15046877 10.1016/j.tins.2004.02.007

[b22] Penttonen M. & Buzsáki G. Natural logarithmic relationship between brain oscillators. Thalamus and Related Systems 2, 145–152 (2003).

[b23] Hoptman M. J. *et al.* Amplitude of low-frequency oscillations in schizophrenia: a resting state fMRI study. Schizophr. Res. 117, 13–20 (2010). 19854028 10.1016/j.schres.2009.09.030 PMC2822110

[b24] Supekar K., Menon V., Rubin D., Musen M. & Greicius M. D. Network analysis of intrinsic functional brain connectivity in Alzheimer's disease. PLoS Comput. Biol. 4, e1000100 (2008). 18584043 10.1371/journal.pcbi.1000100 PMC2435273

[b25] Wang J. *et al.* Disrupted functional brain connectome in individuals at risk for Alzheimer's disease. Biol. Psychiatry 73, 472–481 (2013). 22537793 10.1016/j.biopsych.2012.03.026

[b26] Maxim V. *et al.* Fractional Gaussian noise, functional MRI and Alzheimer's disease. Neuroimage 25, 141–158 (2005). 15734351 10.1016/j.neuroimage.2004.10.044

[b27] Lynall M.-E. *et al.* Functional connectivity and brain networks in schizophrenia. J. Neurosci. 30, 9477–9487 (2010). 20631176 10.1523/JNEUROSCI.0333-10.2010 PMC2914251

[b28] Meng C. *et al.* Aberrant topology of striatum's connectivity is associated with the number of episodes in depression. Brain 137, 598–609 (2014). 24163276 10.1093/brain/awt290

[b29] Jung R. E. & Haier R. J. The Parieto-Frontal Integration Theory (P-FIT) of intelligence: converging neuroimaging evidence. Behav. Brain Sci. 30, 135–154; discussion 154–187 (2007). 17655784 10.1017/S0140525X07001185

[b30] Zalesky A., Fornito A. & Bullmore E. T. Network-based statistic: Identifying differences in brain networks. Neuroimage 53, 1197–1207 (2010). 20600983 10.1016/j.neuroimage.2010.06.041

[b31] Wu F. *et al.* Whiter matter abnormalities in medication-naive subjects with a single short-duration episode of major depressive disorder. Psychiatry Res. - Neuroimaging 191, 80–83 (2011). 21145709 10.1016/j.pscychresns.2010.09.002 PMC3058813

[b32] Seeley W. W. *et al.* Dissociable intrinsic connectivity networks for salience processing and executive control. J. Neurosci. 27, 2349–2356 (2007). 17329432 10.1523/JNEUROSCI.5587-06.2007 PMC2680293

[b33] Harvey P. O. *et al.* Cognitive control and brain resources in major depression: An fMRI study using the n-back task. Neuroimage 26, 860–869 (2005). 15955496 10.1016/j.neuroimage.2005.02.048

[b34] Kaiser S. *et al.* Executive control deficit in depression: Event-related potentials in a Go/Nogo task. Psychiatry Res. - Neuroimaging 122, 169–184 (2003). 12694891 10.1016/s0925-4927(03)00004-0

[b35] Lu Q. *et al.* Impaired prefrontal-amygdala effective connectivity is responsible for the dysfunction of emotion process in major depressive disorder: A dynamic causal modeling study on MEG. Neurosci. Lett. 523, 125–130 (2012). 22750155 10.1016/j.neulet.2012.06.058

[b36] Guilloux J.-P. *et al.* Molecular evidence for BDNF- and GABA-related dysfunctions in the amygdala of female subjects with major depression. Molecular Psychiatry 17, 1130–1142 (2012). 21912391 10.1038/mp.2011.113 PMC3237836

[b37] Hamani C. *et al.* The subcallosal cingulate gyrus in the context of major depression. Biological Psychiatry 69, 301–308 (2011). 21145043 10.1016/j.biopsych.2010.09.034

[b38] Bhagwagar Z. *et al.* Reduction in Occipital Cortex γ-Aminobutyric Acid Concentrations in Medication-Free Recovered Unipolar Depressed and Bipolar Subjects. Biol. Psychiatry 61, 806–812 (2007). 17210135 10.1016/j.biopsych.2006.08.048

[b39] Petty F. GABA and mood disorders: A brief review and hypothesis. J. Affect. Disord. 34, 275–281 (1995). 8550953 10.1016/0165-0327(95)00025-i

[b40] Northoff G. & Bermpohl F. Cortical midline structures and the self. Trends in Cognitive Sciences 8, 102–107 (2004). 15301749 10.1016/j.tics.2004.01.004

[b41] Northoff G. Psychopathology and pathophysiology of the self in depression - Neuropsychiatric hypothesis. Journal of Affective Disorders 104, 1–14 (2007). 17379318 10.1016/j.jad.2007.02.012

[b42] Grimm S. *et al.* Increased self-focus in major depressive disorder is related to neural abnormalities in subcortical-cortical midline structures. Hum. Brain Mapp. 30, 2617–2627 (2009). 19117277 10.1002/hbm.20693 PMC6870821

[b43] Riso L. P. *et al.* Cognitive aspects of chronic depression. J. Abnorm. Psychol. 112, 72–80 (2003). 12653415

[b44] Cuijpers P. *et al.* Psychotherapy for chronic major depression and dysthymia: A meta-analysis. Clinical Psychology Review 30, 51–62 (2010). 19781837 10.1016/j.cpr.2009.09.003

[b45] Fu C. H. Y. *et al.* Neural responses to happy facial expressions in major depression following antidepressant treatment. Am. J. Psychiatry 164, 599–607 (2007). 17403973 10.1176/ajp.2007.164.4.599

[b46] Heller A. S. *et al.* Relationships between changes in sustained fronto-striatal connectivity and positive affect in major depression resulting from antidepressant treatment. Am. J. Psychiatry 170, 197–206 (2013). 23223803 10.1176/appi.ajp.2012.12010014 PMC3563751

[b47] Delaveau P. *et al.* Brain effects of antidepressants in major depression: A meta-analysis of emotional processing studies. J. Affect. Disord. 130, 66–74 (2011). 21030092 10.1016/j.jad.2010.09.032

[b48] Smith S. M. *et al.* Network modelling methods for FMRI. Neuroimage 54, 875–891 (2011). 20817103 10.1016/j.neuroimage.2010.08.063

[b49] Oldfield R. C. The assessment and analysis of handedness: The Edinburgh inventory. Neuropsychologia 9, 97–113 (1971). 5146491 10.1016/0028-3932(71)90067-4

[b50] Hamilton M. A rating scale for depression. J. Neurol. Neurosurg. Psychiatry 23, 56–62 (1960). 14399272 10.1136/jnnp.23.1.56 PMC495331

[b51] Friston K. J., Williams S., Howard R., Frackowiak R. S. & Turner R. Movement-related effects in fMRI time-series. Magn. Reson. Med. 35, 346–355 (1996). 8699946 10.1002/mrm.1910350312

[b52] Yan C. G. *et al.* A comprehensive assessment of regional variation in the impact of head micromovements on functional connectomics. Neuroimage 76, 183–201 (2013). 23499792 10.1016/j.neuroimage.2013.03.004 PMC3896129

[b53] Fox M. D., Zhang D., Snyder A. Z. & Raichle M. E. The global signal and observed anticorrelated resting state brain networks. J. Neurophysiol. 101, 3270–3283 (2009). 19339462 10.1152/jn.90777.2008 PMC2694109

[b54] Murphy K., Birn R. M., Handwerker D. A., Jones T. B. & Bandettini P. A. The impact of global signal regression on resting state correlations: Are anti-correlated networks introduced? Neuroimage 44, 893–905 (2009). 18976716 10.1016/j.neuroimage.2008.09.036 PMC2750906

[b55] Makris N. *et al.* MRI-Based topographic parcellation of human cerebral white matter and nuclei II. Rationale and applications with systematics of cerebral connectivity. Neuroimage 9, 18–45 (1999). 9918726 10.1006/nimg.1998.0384

[b56] Percival D. B. & Walden A. T. *Wavelet Methods for Time Series Analysis*. Communications in Statistics Simulation and Computation 10, 594 (2000).

[b57] Bassett D. S. *et al.* Dynamic reconfiguration of human brain networks during learning. Proc. Natl. Acad. Sci. U. S. A. 108, 7641–7646 (2011). 21502525 10.1073/pnas.1018985108 PMC3088578

[b58] Watts D. & Strogatz S. Collective dynamics of “small-world”networks. Nature 393, 440–442 (1998). 9623998 10.1038/30918

[b59] Rubinov M. & Sporns O. Complex network measures of brain connectivity: Uses and interpretations. Neuroimage 52, 1059–1069 (2010). 19819337 10.1016/j.neuroimage.2009.10.003

[b60] Zalesky A. *et al.* Disrupted axonal fiber connectivity in schizophrenia. Biol. Psychiatry 69, 80–89 (2011). 21035793 10.1016/j.biopsych.2010.08.022 PMC4881385

