# Supplementary material for: Frequency Dependant Topological Alterations of Intrinsic Functional Connectome in Major Depressive Disorder
Source: Sci Rep. 2015 Apr 9;5:9710. doi: 10.1038/srep09710 (PMC5155486; doi:10.1038/srep09710)
Supplement: Supplementary Information — Definition of topological metrics [file srep09710-s1.doc]

**Frequency Dependant Topological Alterations of Intrinsic Functional Connectome in Major Depressive Disorder**

**Authors:** Qinghua Luo^1†^, Zhou Deng^2,3†^, Jingxue Qing^2,3^, Dongtao Wei^2,3^, Lingli Cun^2,3^, Jiang Qiu^2,3^, Glen Hitchman^2,3^, Peng Xie^1*^

†Equal contribution

*Corresponding author

# Affiliations:

# ^1^The First Affiliated Hospital of Chongqing Medical University, Chongqing, China

# ^2^Key laboratory of cognition and personality (SWU), Ministry of Education, Chongqing 400715, China

# ^3^Department of psychology, Southwest University, Chongqing 400715, China

**Corresponding To**:

Professor Peng Xie

Department of Neurology

The First Affiliated Hospital of Chongqing Medical University

Institute of Neuroscience, Chongqing Medical University

No.1 Yixueyuan Road, Yuzhong District, Chongqing, 400016, China

Tel: +86-23-68485490

Fax: +86-23-68485111

E-mail: xiepeng@cqmu.edu.cn

Professor Jiang Qiu

Department of Psychology

Southwest University

Chongqing 400715, China

Tel: +86-23-68367942

E-mail:qiu318@swu.edu.cn

Definition of topological metrics

Clustering coefficient

The clustering coefficient captures cliquishness of a network. The clustering coefficient ($C_{i}$) for a node $i$ is calculated as ^1^:

Where $\mathop{w}\limits^{\sim} _{ij}$ is the scaled (by the sum of whole existing edge intensities) pairwise wavelet correlation coefficient between node $i$ and node $j$ in a connected subgragh ($\mathrm{G}_{\mathrm{i}}$), $k_{i}$is the weighted degree of node $i$. This scaling has the advantage of being consistent with binary network and ensure identical level of connectivity strength across all subjects^1^. Globally, clustering coefficient of a given network was average clustering coefficient of all nodes.

Characteristic path length

The characteristic path length indicates the routing efficiency between any two regions. It is calculated as the minimum path length between any two nodes to allow for possible isolated nodes:

$$\frac{1}{L}=\frac{1}{N\left(N-1\right)}\sum_{i\neq j\in G} \frac{1}{d_{ij}}$$

Where N is the number of nodes, $d_{ij}$denotes the minimum path length between any two nodes ($i$ and $j$).

Small worldness

The small worldness ($\sigma$) is qualitatively defined as highly clustering while maintaining relatively low characteristic path length. Quantitatively, it is defined as:

$$\sigma=\frac{C/C_{R}}{L/L_{R}}$$

Where $C_{R}$ is the corresponding clustering coefficient of random network (see below) and $L_{R}$the characteristic path length. $\sigma$ of network with small worldness usually is larger than 1^2,3^.

Global efficiency

The global efficiency is defined as the geometry mean of inverse minimum path length across all pair of nodes and further reflects information integration within a network. Compared with characteristic path length, global efficiency is mainly affected by short-range path^2^.

Nodal centrality

Nodal centrality includes degree centrality as well as betweenness centrality here. Degree centrality of a given node is calculated as the sum of weighted edges connected to it. Betweenness centrality of a given node is defined as the reciprocal of all minimum path length that pass through the node. Betweenness centrality identifies most passed-by nodes while degree centrality most connected nodes^2^.

Random network

Random network was generated with the constraint that it should have identical number of nodes, edges and also the degree distribution with the real brain network ^4^. Here, we calculated the mean topological metric (global and regional metric mentioned above) across 100 random weighted networks.

**References**

1. Onnela, J. P., Saramäki, J., Kertész, J. & Kaski, K. Intensity and coherence of motifs in weighted complex networks. *Phys. Rev. E - Stat. Nonlinear, Soft Matter Phys.* **71,** (2005).

2. Rubinov, M. & Sporns, O. Complex network measures of brain connectivity: Uses and interpretations. *Neuroimage* **52,** 1059–1069 (2010).

3. Watts, D. & Strogatz, S. Collective dynamics of “small-world”networks. *Nature* **393,** 440–442 (1998).

4. Sporns, O. & Zwi, J. D. The small world of the cerebral cortex. *Neuroinformatics* **2,** 145–162 (2004).
